# Supplementary material for: Multimodal imaging and functional analysis of the chick NMDA retinal damage model
Source: PLoS One. 2021 Sep 7;16(9):e0257148. doi: 10.1371/journal.pone.0257148 (PMC8423281; doi:10.1371/journal.pone.0257148)
Supplement: S1 File — (ZIP) [file pone.0257148.s004.zip › SD-OCT/C200727/010/D21-Pwk4/C200727-010_OD_V_14X14_0_REGAVG0000122/Manual_PreDefineFiveByFiveGridRectangularRetina_1.pdf]

# Five By Five Grid Template Report

| Patient ID | C200727 | Name | See InVivoVue |
|---|---|---|---|
| Exam Date | See InVivoVue | Exam Time | See InVivoVue |
| Eye | OD | Averaged | N/A |
| Depth (mm) | 1.65 | Depth Samples | 1024 |
| Pitch (um/pix) | 35.000 x 35.000 x 1.608 | Refractive Index | See InVivoVue |
| Scan Type | Rectangular 14.000mm x 14.000mm x 1.647mm ,<br>400 x 400 x 1024 | | |

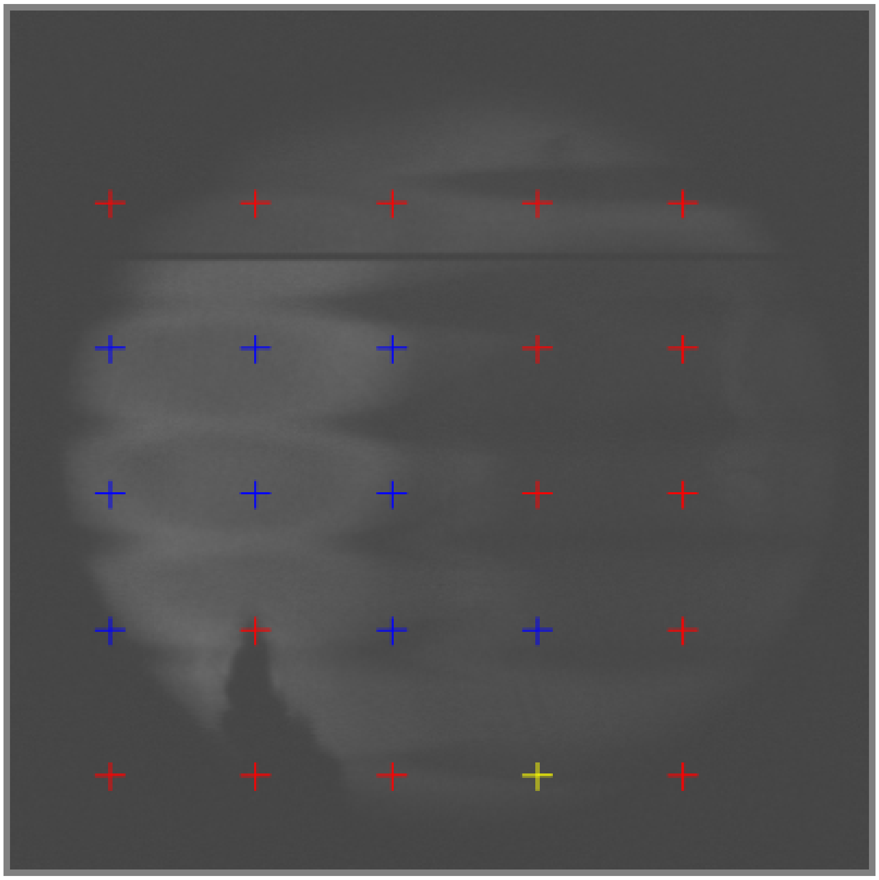

## Thickness Metrics

| Analysis | Average(mm) | Minimum(mm) | Maximum(mm) | STDEV(mm) |
|---|---|---|---|---|
| IRNFL - RPE | 0.2832 | 0.2702 | 0.3039 | 0.0111 |
| IRNFL - ORNFL | 0.0384 | 0.0162 | 0.0537 | 0.0125 |
| ORNFL -  OPL | 0.1650 | 0.1510 | 0.1903 | 0.0133 |
| OPL  -  ETPRS | 0.0615 | 0.0506 | 0.0674 | 0.0051 |

Q:\Bioptigen\Data\images\site3000254\image\2020.08.24\C200727-010_OD_V_14X14_0_REGAVG0000122.OCT

2/24/2021 12:22:08 PM
